# Effect of Sonication and Ceria Doping on Nanoparticles Fabricated by Laser Marker Ablation of Ti in Water

**DOI:** 10.3390/nano13152201

**Published:** 2023-07-28

**Authors:** Huixing Zhang, Xiaowen Qi, Chengling Liu, Xiaojie Chen, Chao Teng, Yang Luo, Chenrui Wang, Hui Jiang, Hongtao Cui, Ji Dong

**Affiliations:** 1 School of Mechanical Engineering, Tianjin Sino-German University of Applied Sciences, Tianjin 300350, China; 2 Department of Materials Science, School of Civil Engineering, Qingdao University of Technology, Qingdao 266520, China; qixiaowen150@126.com (X.Q.); chenxiaojie82022@163.com (X.C.); 15870028528@163.com (Y.L.);

**Keywords:** nanoparticles, laser ablation in water, sonification, ceria

## Abstract

By employing the laser marker fast ablation technique in water, combined with the innovative inclusion of sonication, we successfully developed Ti-based nanoparticles with improved characteristics. sonication increased the nanoparticle concentration in the colloid, reduced nanoparticle size, and also narrowed size distribution. Our findings also provide valuable insights into the influence of laser parameters, such as wavelength and fluence, on nanoparticle properties. UV laser led to small nanoparticles compared with 1064 nm laser. Additionally, high laser fluence appeared to increase the ablated particle size until a plateau fluence at 28.5 J/cm^2^; at 38 J/cm^2^, the particle size decreased. Notably, all synthesized particles exhibited a regular spherical shape, as confirmed by energy dispersive X-ray spectroscopy (EDS) mapping, which also indicated that the majority of Ti-based particles were in an oxidized state. Additionally, the presence of rutile TiO_2_ in the particles was further confirmed by X-ray diffraction (XRD) analysis. Ceria doping Titania nanoparticles was also attempted.

## 1. Introduction

TiO_2_, known for its affordability, stability, and abundance on Earth, exhibits excellent properties such as UV light absorption and water splitting capabilities [1,2,3]. However, its application is limited by its large bandgap of 3–3.2 eV, which restricts its absorption to only UV spectrum [4]. TiO_2_ nanoparticles (NPs) have been utilized to reduce its bandgap and shift its color [5]. Therefore, TiO_2_ NPs have found wide-ranging applications in hydrogen generation, solar cells, and photocatalyst to degrade organic pollutants [3,4,5,6,7]. Especially for electrochemical nitrate-to-ammonia conversion, Ti-based NPs have been widely acknowledged as the most promising catalysts [8].

Among the various methods available for synthesizing TiO_2_ NPs, pulsed laser ablation in liquid (PLAL) stands out as an effective and environmentally friendly approach. This technique offers numerous advantages, including easy production and extraction, simplified synthetic procedures without the need for multiple steps, reduced reaction time, absence of reducing agents, enhanced laboratory safety, and low toxicity [9,10,11,12]. Upon laser irradiation of the metal target, the radiation is absorbed by the target surface electrons, leading to instantaneous energy transfer to the lattice [10]. The high power density results in explosive vaporization of the local surface, creating a plasma plume with a volume defined by the laser spot size and a height of approximately 100 nm within a few hundred picoseconds [13]. The rapid volume expansion induces a shockwave both in the target and the liquid, causing a phase transition in the target. The interaction between plasma and the liquid vaporizes the liquid, initiating a cavitation bubble primarily composed of liquid vapor. This cavitation bubble encloses a plasma layer with a thin transient metal layer on both sides. Thus, each pulse generates a structure consisting of a bubble cap, a transient thin metal layer between the cap and the plasma, the plasma layer itself, and another transient thin metal layer between the plasma and the target surface. The NPs are mainly expulsed from the bursting of the cavitation bubble. The cavitation bubble grows and collapses typically within the time scale of hundred of microseconds, which becomes a limiting process in NP generation. Furthermore, reflection from the water/air as well as water/target interfaces, refraction, and self-focusing in water lead to a decrease in the effective energy deposited on the target surface compared to the nominal output power [14]. Therefore, the production rate is low. The scattering of the cavitation bubble, the shielding effect of the plasma layer, and the absorption and re-ablation of the ablated particles reduce further the effective energy deposited on the target [13]. Stirring a flow liquid has been found to assist in removing generated bubbles and particles compared with a static liquid, thereby improving production [15]. Attempts to increase production through the application of electrical, magnetic, and thermal fields revealed that only a magnetic field proved effective by increasing the kinetic energy of charged electrons and the plasma density or accelerating plasma breakdown due to the stirring effect [10,16]. High laser-scanning speed has been reported to increase production dramatically as the interpulse distance becomes much larger than the bubble size [14]. Under such conditions, the cavitation bubble is bypassed, and the bypassed bubble and the enclosed plasma layer cannot shield the following laser pulse [14]. In summery, circumventing the bubble for the following pulse, accelerating the break down of the bubble, increasing the plasma density, removing the generated bubbles and particles have proved to be effective strategies to increase production rate of PLAL. For further scale up, remote control of PLAL has been demonstrated [17] and the application of an electrical field has been found instrumental in confining the plasma plume as well as collecting particles [18]. Apart from the self-focusing and filamentation effect [13], the penetration depth per pulse for ultrashort laser such as fs and ps lasers is relatively shallow, two orders of magnitude lower than that of ns lasers [19], resulting in lower ablation efficiency [13]. In this regard, low-power, inexpensive, and compact ns lasers have shown promise in terms of ablation efficiency, with a factor of 8, which is higher than that of high-end ps lasers [13]. Moreover, laser ablation in water enables the production of small NPs with a narrow size distribution compared to other liquids [9], exhibiting a log-normal size distribution [20].

Laser marker is cheap and facile to operate with high energy density and fast scanning speed, which is beneficial to bypass the cavitation bubble and achieve high production. In this work, a laser marker was adopted to ablate Ti target in distilled water to produce NPs. Meanwhile, sonication was introduced to laser NP production and its effect on NPs was investigated, which may accelerate the collapse of the bubble and is compatible with other production enhancement methods.

Ceria-doped Titania NPs have been utilized as a promising efficient drug delivery system [21]. Ceria and Titania composites have also been active catalysts and photocatalysts for wide applications and have drawn wide attention [22,23,24,25,26,27,28]. In this study, the doping of ceria in titania was attempted using PLAL, adding to the exploration of this composite material.

## 2. Materials and Methods

**Figure 1:**
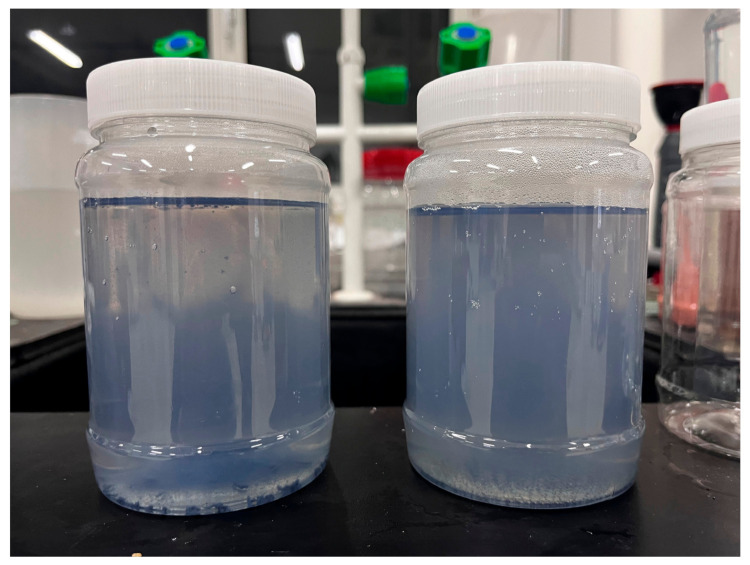
Typical colloids in the storage jars produced with (**right**) and without (**left**) sonication.

A measuring cylinder with a 1% volume precision was used to measure fixed volume ~500 mL distilled water into the supersonic bath tank. A 100 × 100 × 5 mm 99.995% Ti block was immersed in the water ~10 mm under the water surface and ablated by a pulsed optical fiber laser marker (YLP-M30, Qingdao, China) with wavelength at 1064 nm, 40 KHz repetition rate, pulse duration of 100 ns, and 30 W nominal outpower. The laser beam was focused onto the target using a scanning head equipped with a focusing lens, resulting in a focused beam size of approximately 50 μm in diameter. At nominal output power, the laser fluence was 38 J/cm^2^. However, due to reflection, refraction, and non-linear effects in water, the effective fluence deposited on the target surface would be much reduced. The laser ablation was conducted in raster scan mode with the laser head move controlled by a computer. The laser ablation was taken with and without sonication for comparison. The sonication was carried out at a frequency of 40 KHz, matching the laser repetition rate. Laser fluence was also varied at different levels: 7.6 J/cm^2^, 19 J/cm^2^, 28.5 J/cm^2^, and 38 J/cm^2^. The scanning rate was 10 m/s with line spacing of 0.06 mm and scanning duration was fixed 0.5 h for all processing conditions. In addition to the pulsed optical fiber laser marker, a pulsed UV laser marker (XC-XMU5W, Qingdao, China) with a wavelength of 355 nm, a repetition rate of 40 KHz, and a nominal output power of 5 W was also employed. The raster scan settings were the same as those used for the optical fiber laser, except for the pulse duration, which was set at 1 microsecond. At such settings, the laser fluence was about 6 J/cm^2^. After each round of TiO_2_ NPs fabrication, the entire fluid was transferred into a standard jar for storage. Figure 1 shows typical colloids synthesized with and without sonication in the storage jars. Sonication resulted in deep color which suggested high NPs concentration and less large precipitates at the bottom.

In order to dope ceria into Ti-based particles, ceria powders with a diameter of 10 μm were dispersed into the water prior to laser ablation. The mass of the powder was varied for comparative analysis, specifically at 0.025 g and 0.1 g measured by a micro-balance, corresponding to concentrations of 50 ppm and 200 ppm in the water medium. All the laser ablation parameters were the same for both.

The liquid sampling drop from the stored colloids was dispensed onto a Al substrate by a pipette followed by drying in an oven for measurement. Scanning electron microscope (SEM) images were taken in Sigma300 from CARL ZEISS (Jena, Germany) with a resolution of 1 nm for extracting the morphology of laser ablated NPs. Energy dispersive X-ray spectroscopy (EDS) mappings were also performed to extract composition distribution over the selected regions. EDS of selective points was also measured. Bruker D8 Advance was adopted to extract X-ray diffraction (XRD) spectra of the colloidal solutions. A laser scanning confocal microscope with the model number OLS5100 from Olympus Corporation (Tokyo, Japan) was employed to examine 3D profiles of the ablated target surface with and without sonication to obtain the ablated volumes for comparison of the production rates. The ablated volumes were extracted from the attached software. The UV-vis spectrophotometer (UV2600i by Shimadzu, Kyoto, Japan) was used to measure the transmission of the colloidal solutions in the wavelength range of 190 nm to 1100 nm. Nanoparticle size distributions were analyzed by means of a dynamic light scattering analyzer (Malvern Zetasizer Nano ZS, Malvern, UK).

## 3. Results and Discussion

**Figure 2:**
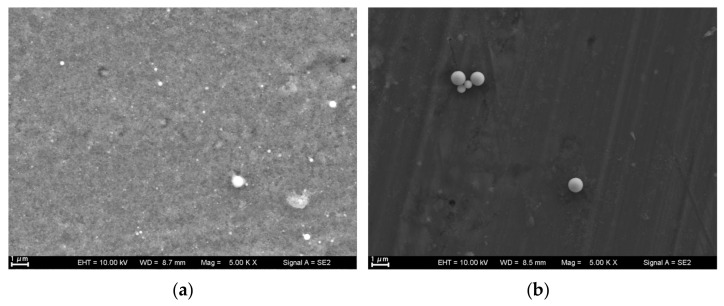

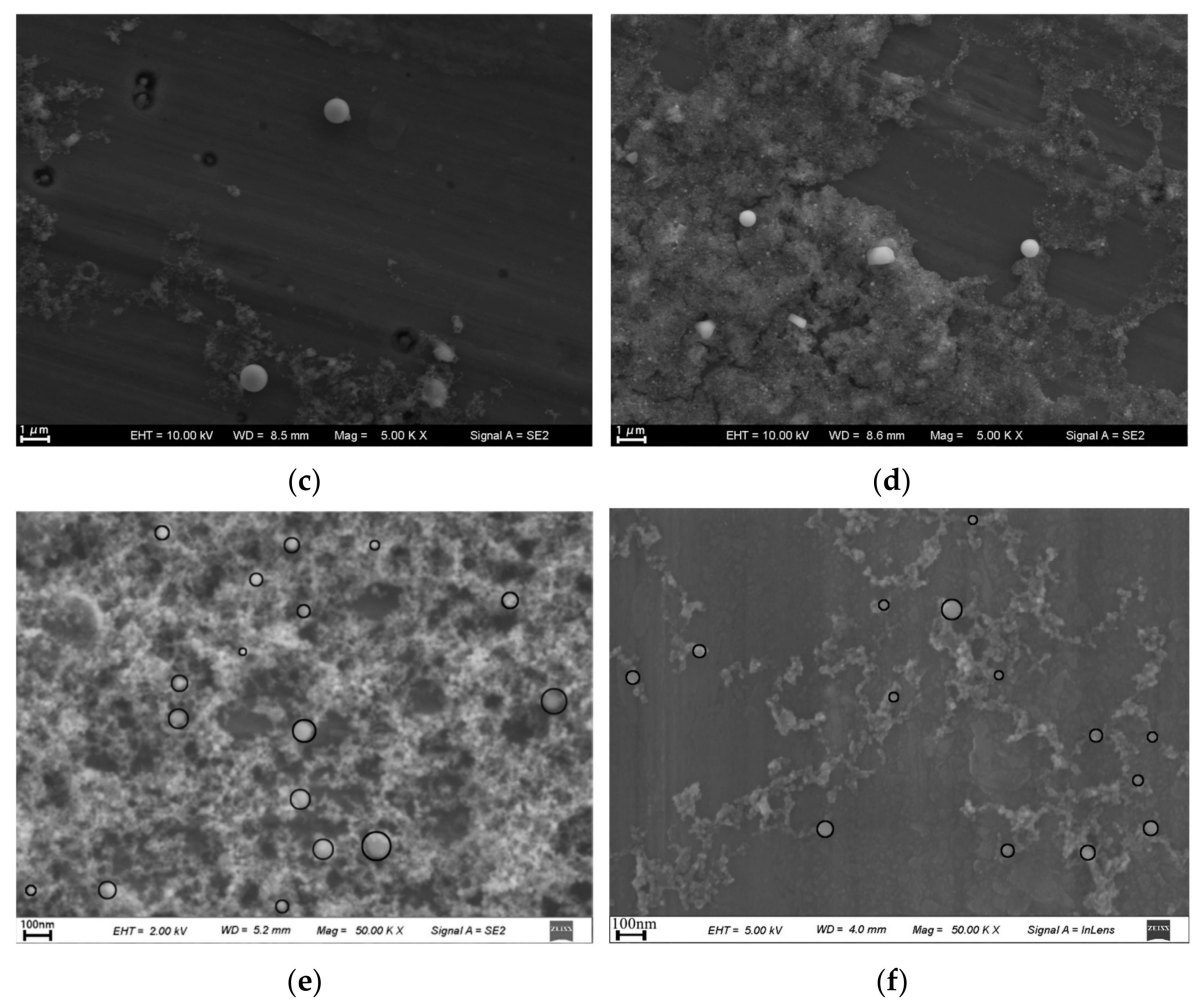
SEM of 1064 nm laser-produced nanoparticles at varying fluence, with and without sonication: (**a**) 7.6 J/cm^2^ without sonication, (**b**) 19 J/cm^2^ without sonication, (**c**) 28.5 J/cm^2^ without sonication, (**d**) 38 J/cm^2^ without sonication, and (**e**) 7.6 J/cm^2^ without sonication (**f**) 7.6 J/cm^2^ with sonication.

**Figure 3:**
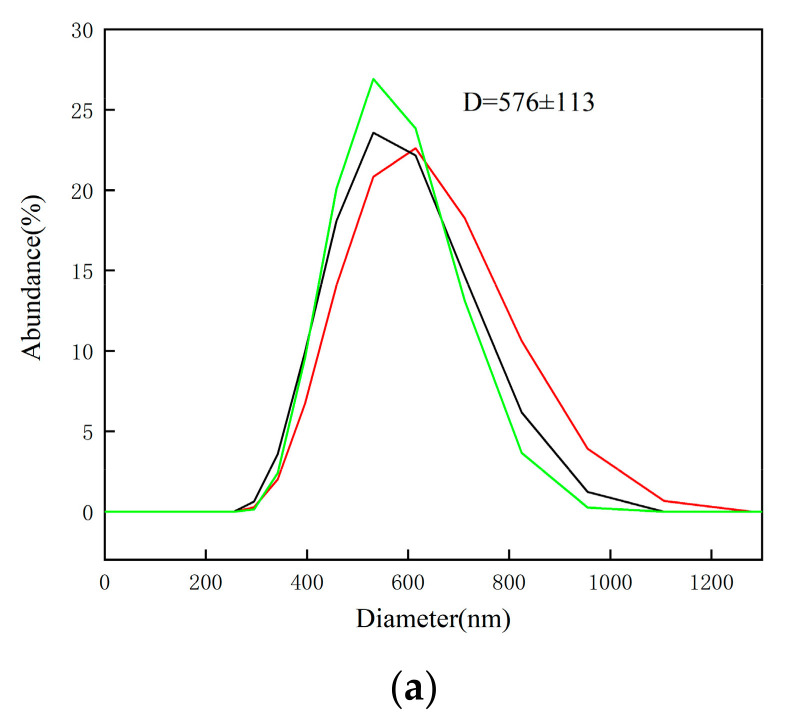

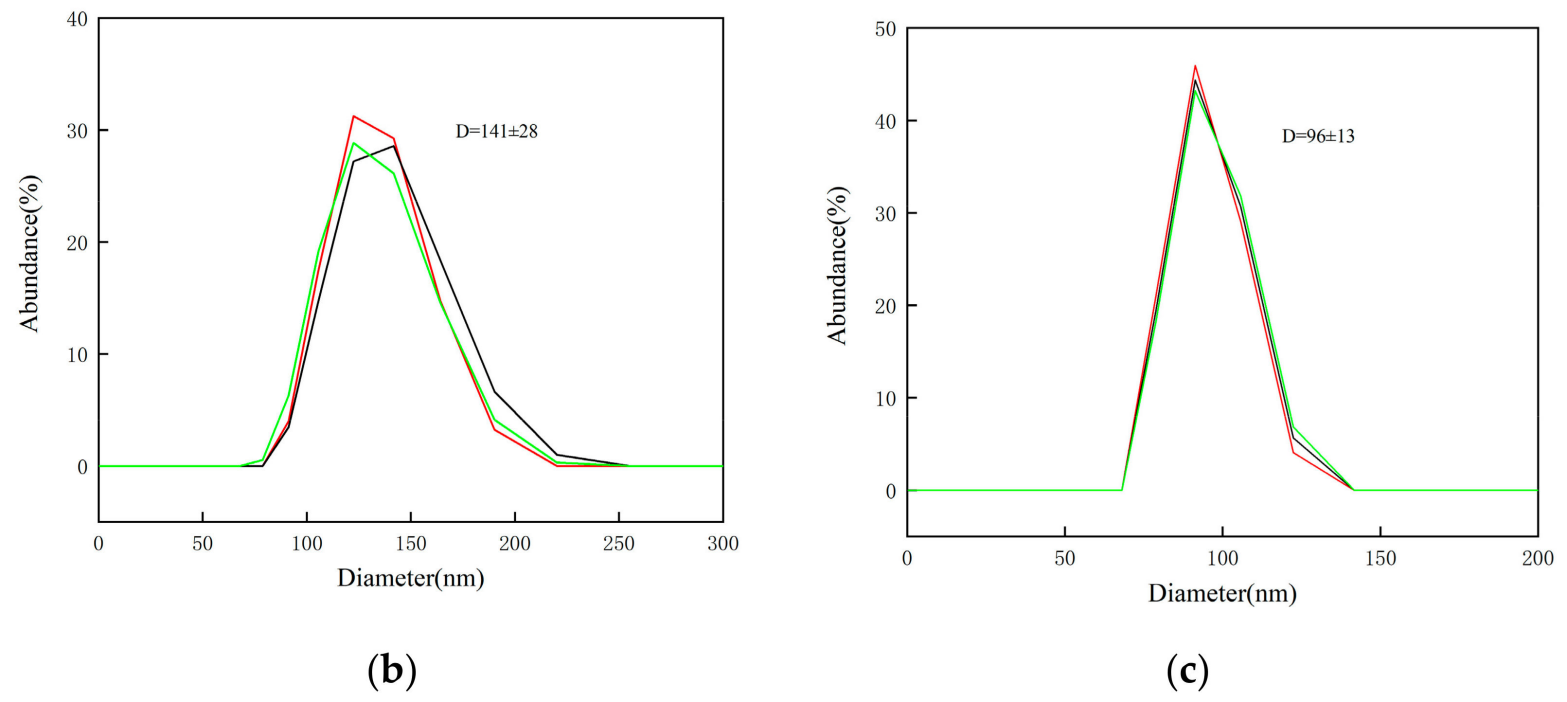
(**a**). Dynamic light scattering (DLS) spectra of particles produced at 38 J/cm^2^ without sonication. (**b**). DLS spectra of particles produced at 7.6 J/cm^2^ without sonication. (**c**). DLS spectra of particles produced at 7.6 J/cm^2^ with sonication. Three measurements were taken for each colloidal solution to ensure precision and reproducibility.

**Figure 4:**
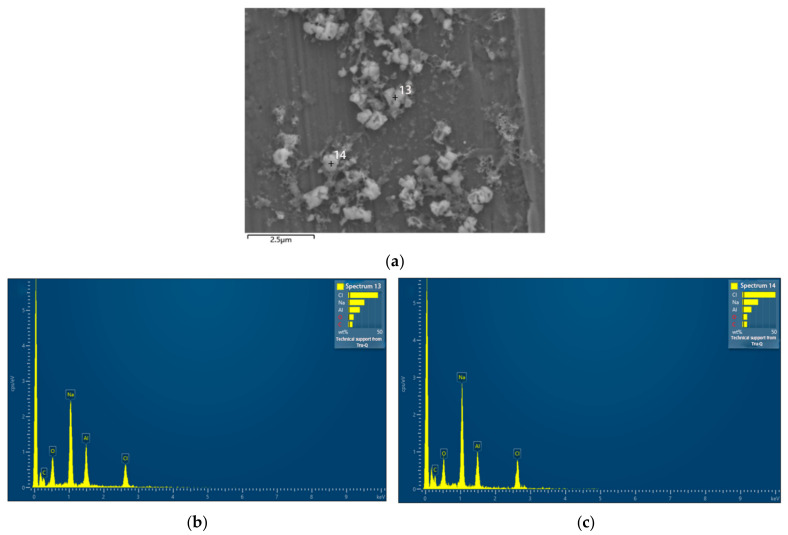
(**a**) SEM image of a different region of the same sample presented in Figure 2f. The crossings labeled with numbers 13 and 14 indicate the selected points for EDS measurements. (**b**) EDS result of point 13 in (**a**). (**c**) EDS result of point 14 in (**a**).

SEM images of the laser-produced Ti particles at varying laser fluences with and without sonication are shown in Figure 2. The images demonstrate that all particles were regularly spherical. At a low fluence of 7.6 J/cm^2^, the particle size exhibited a wide distribution, with the majority of particles appearing as NPs. Particle size increased with laser fluence until the plateau fluence 28.5 J/cm^2^ followed by a size decrease at 38 J/cm^2^. This decrease in particle size at higher fluence can be attributed to the further breakdown of the ablated particles under the influence of high laser energy. Upon close examination at high magnification in Figure 2e (at 7.6 J/cm^2^), it can be observed that the laser fluence produced NPs with a majority well below 100 nm, and some even as small as approximately 20 nm. Figure 3a presents size distribution of particles produced at 38 J/cm^2^, which is consistent with Figure 2d. Figure 3a demonstrates the maximum abundance of particles occurs around 600 nm, a size range that aligned with ~600 nm sized particles shown in Figure 2d. Figure 3b,c show two additional DLS results for colloidal solutions processed at 7.6 J/cm^2^ without and with sonication, respectively. Sonication generally produced smaller particles with narrower size distribution, corroborating the findings depicted in Figure 2e,f. It is essential to acknowledge a slight discrepancy between the particle distribution data obtained from DLS spectra and SEM images. SEM provides insights into only a relatively small, localized portion of the particle distribution and, as a result, may sometimes contradict the overall particle distribution observed through DLS analysis. These disparities can be attributed to the inherent limitations of each technique, wherein SEM offers higher resolution imaging of specific small portion of particles, while DLS provides a more comprehensive characterization of the entire colloidal solution. It is important to note that only the spherical particles in Figure 2e,f were Ti-based particles, which are highlighted with circles. The non-spherical particles were contaminants, which would be verified in Figure 4.

Figure 2e took much attention due to its low concentration and sparse distribution of particles. Conversely, Figure 2f was easier to capture as the concentration of particles was higher. The stirring effect of sonication increased the chances of laser interaction with the particles, leading to further breakdown and size reduction. Sonication also likely played a role in splitting bubbles and expulsed melts generated during the laser ablation process, contributing to particle size reduction [10]. Furthermore, sonication might potentially expedite the collapse of the cavitation bubble and plasma plume [10], enhancing the particle production rate. This effect arose from the operating frequency of sonication, which was nearly one order magnitude higher than that of cavitation bubble burst. Given that both the cavitation bubble and the plasma layer exist in a metastable state, a minor perturbation such as high-frequency sonication is sufficient to disrupt this state, resulting in the expulsion of NPs. This could explain why sonication led to a deep-colored colloid in Figure 1, indicating a higher concentration of particles.

Figure 4 shows a SEM image of a different region of the same sample presented in Figure 2f and EDS spectra of selected non-spherical particles. Figure 4 suggests that the non-spherical particles were mostly salts (Na and Cl peaks) or organic contaminants (C peak), which might come from fingerprint residuals during sample preparation for measurement and airborne pollutants.

**Figure 5:**
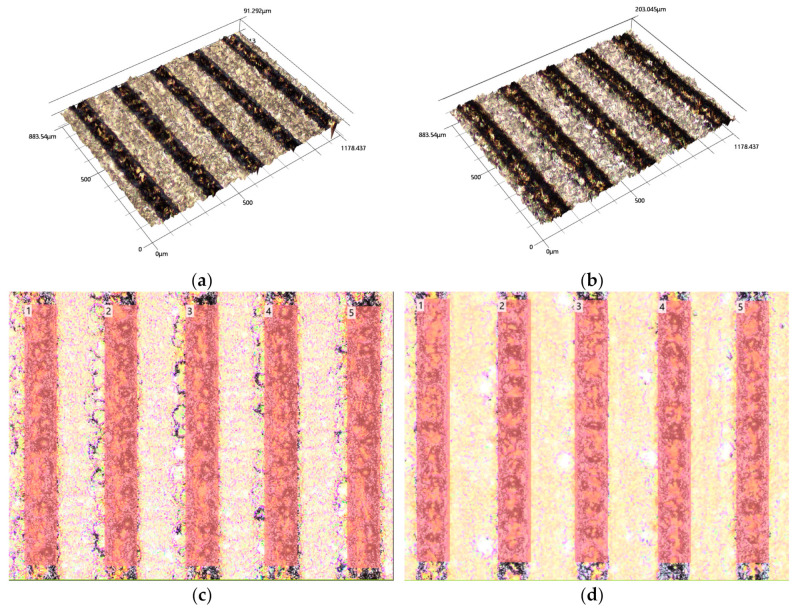
Laser confocal images of representative ablated target surfaces with and without sonication. (**a**). A 3D profile of sample without sonication. (**b**). A 3D profile of sample with sonication. (**c**). A 2D profile of sample without sonication. The marked regions are selected regions for volume extraction 100 μm × 800 μm each. (**d**). A 2D profile of sample with sonication. The marked regions are selected regions for volume extraction 100 μm × 800 μm each.

Figure 5 showcases confocal images of treated targets with and without sonication. The darker regions represent the ablated areas, while the lighter regions represent the untreated intact parts. The introduction of sonication resulted in deep, ablated channels and a pronounced contrast between the ablated and intact regions. The Z dimension values further indicate that sonication led to a larger ablation volume and a higher production rate. Specifically, the ablated volumes of the regions marked in Figure 5c,d were measured to be 0.46 mm^3^ and 0.26 mm^3^, respectively. These findings align consistently with the observations presented in Figure 1. It is important to note that the observed ablated channels in our study differed from the craters described in the literature. However, the objective of this measurement was consistent with the literature, which aimed to assess the production rate of nanoparticles [20,29].

**Figure 6:**
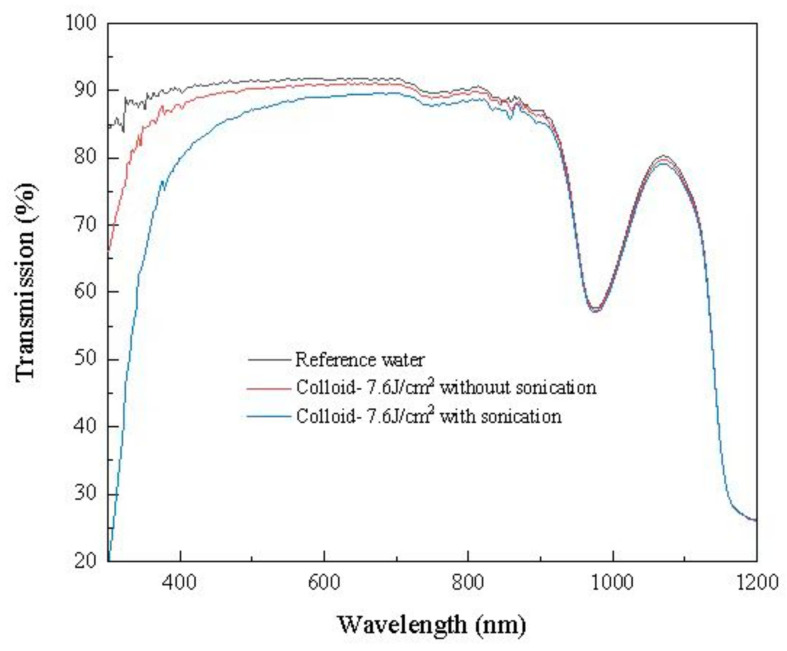
Transmission of reference water and colloidal solutions underwent laser ablation at a fluence of 7.6 J/cm^2^ with and without sonication.

Figure 6 shows transmission of reference water and colloidal solutions produced with and without sonication. In comparison to the reference water, the transmission of colloidal solutions decreased thanks to scattering, reflection, and absorption of the nanoparticles dispersed within them. Sonication resulted in a further reduction in transmission. This can be attributed to the enhanced production rate of nanoparticles facilitated by sonication, a phenomenon consistent with the findings presented in Figure 1 and Figure 5.

**Figure 7:**
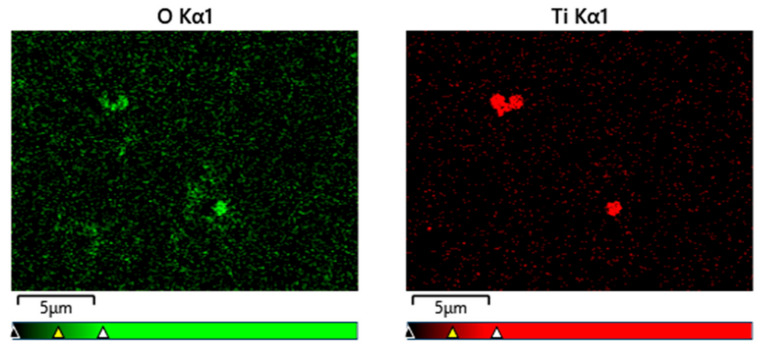
EDS mapping of Figure 2b.

Figure 7 presents EDS mapping of the particles depicted in Figure 2b and it indicates that the majority of the Ti-based NPs were largely oxidized, indicating the possible presence of TiO_2_. This oxide was the reaction product between highly active ablated Ti and O in water. O and Ti signal did not exactly match the spherical particles because the signal accumulation time was not adequate.

**Figure 8:**
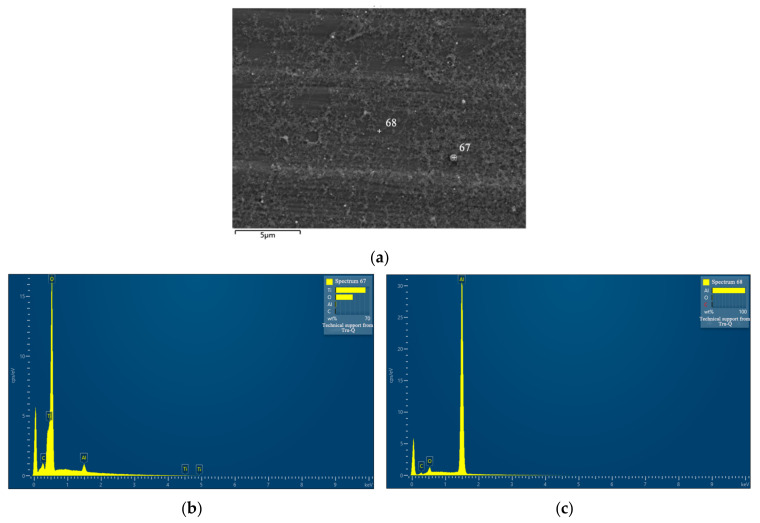
(**a**) SEM image of particles produced from a representative ablation. The crossings labeled with numbers 67 and 68 indicate the selected points for EDS measurements. (**b**) EDS result of point 67 in (**a**). (**c**) EDS result of point 68 in (**a**).

Figure 8 depicts EDS analysis of a particle from a representative ablation and the Al substrate. The crossings labeled with numbers 67 and 68 represent the selected particle and the Al substrate, respectively, for EDS measurement. The C/O/Al atomic ratio for Al substrate was found to be 2.3:2.4:95.3. As for the selected particle, the C/O/Al/Ti atomic ratio was determined to be 1.7:61.3:1.6:35.4, where C was mainly attributed to airborne pollutants and may be bonded with O. EDS typically has a detection depth in the range of microns. Additionally, Al forms a very thin native protective oxide layer on its surface, usually at the level of a few nanometers [30]. Due to this thin oxide layer, the majority of Al-bonded oxygen is primarily associated with surface-bound Al. EDS also has its detection resolution generally ~1% [31]. Consequently, the atomic concentration of O bonded to Al and C was assumed to be relatively constant for both Figure 8b,c, estimated to be approximately 2% based on measurements. As a result, the atomic ratio between O bonded with Ti and Ti was determined to be 59.3:35.4, equivalent to 1.68. This finding indicates insufficient oxidation of Ti, with approximately 16% of the metal remaining unoxidized. Additionally, the observation suggests a core–shell structure of the particle, featuring a TiO_2_ shell encompassing a pure Ti core.

**Figure 9:**
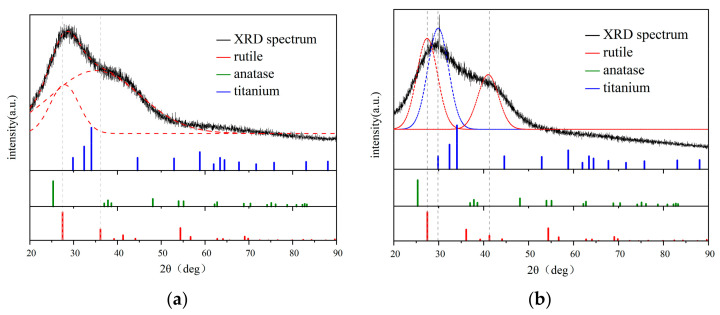
XRD spectrum of colloidal solutions produced at different fluences. (**a**) 19 J/cm^2^. The spectrum was deconvoluted into two rutile peaks (red dotted lines) by fitting using Origin. (**b**) 28.5 J/cm^2^. The spectrum was deconvoluted into two rutile peaks and a Ti peak (blue dotted line) by fitting using Origin.

Figure 9 shows XRD spectra of colloidal solutions, which were fitted using Origin. It indicates the presence of rutile nano-crystal. According to Scherrer equation [32]:

(1) D=Kλ/(βcosθ)

where D is the particle diameter, *K* is constant, λ is the wavelength of X-ray, 0.154 nm, β is full width half maximum (FWHM), θ is the diffraction angle. The narrow, fitted red peaks observed in Figure 9a,b provide valuable insights into the nano-crystal sizes of approximately 1 nm and 1.4 nm, respectively, for rutile. In contrast, the sharp Ti peak represented by the black line in Figure 9b, as opposed to the fitted blue one, corresponds to a larger nano-crystal size of approximately 5 nm for Ti. These calculated sizes are notably smaller than the particles depicted in Figure 2b,c.

This intriguing observation suggests that the particles’ outer shell is likely composed of amorphous Ti oxide, while the core contains a small amount of nano-rutile and nano-Ti. This structural arrangement may explain the significant difference in size between the crystalline structures observed in Figure 9 and the larger particles shown in Figure 2b,c. Furthermore, it is important to recognize that stress-induced diffraction peak broadening during the particle formation process may provide an alternative explanation for the observed phenomenon. The sudden changes in pressure and temperature experienced during particle formation can induce stress, leading to diffraction peak broadening, which is beyond the scope of application of Scherrer equation.

**Figure 10:**
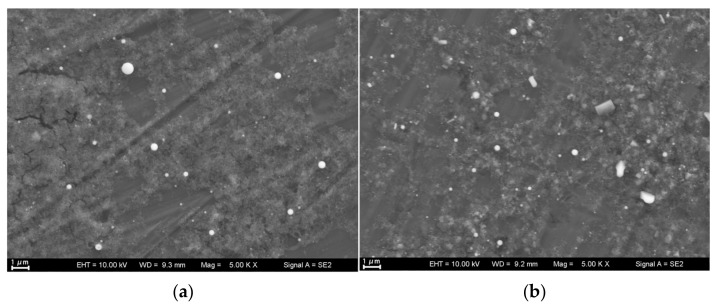
SEM of laser-produced nanoparticles by UV laser marker, without (**a**) and with (**b**) sonication.

**Figure 11:**
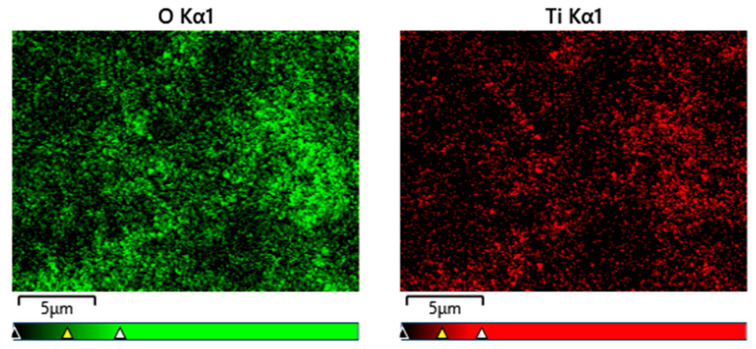
EDS mapping of Figure 10b.

Figure 10 demonstrates that the implementation of the UV laser generally led to smaller particle size compared to its 1064 nm counterpart. Sonication proved, once again, to lead to a reduction in particle size and a narrow size distribution, as is evident from the comparison of the sonicated and non-sonicated samples. It is worth noting that sonication operated at the same frequency as the laser, which could potentially enhance the ablation of particles. Figure 11 illustrates EDS mapping of particles depicted in Figure 10b. It indicates that the majority of the nano-particles were predominantly oxidized, confirming the presence of TiO_2_. It is worth noting that only the spherical particles corresponded to Ti-based particles, as highlighted in the mapping results.

**Figure 12:**
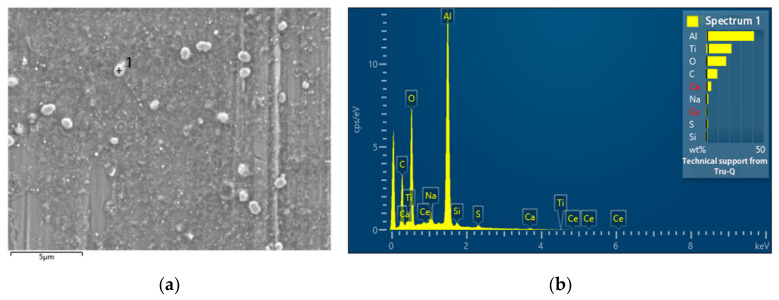
(**a**) SEM image of particles produced with 0.025 g ceria. (**b**) EDS spectrum of selected particle in (**a**). The crossing labeled with number 1 indicate the selected particle for EDS measurement.

Figure 12 presents EDS spectrum of a selected particle produced with 0.025 g ceria. It suggests the successful doping of ceria into titania particle. The Al peak was from the substrate, as EDS detection depth was in a few micrometers level.

**Figure 13:**
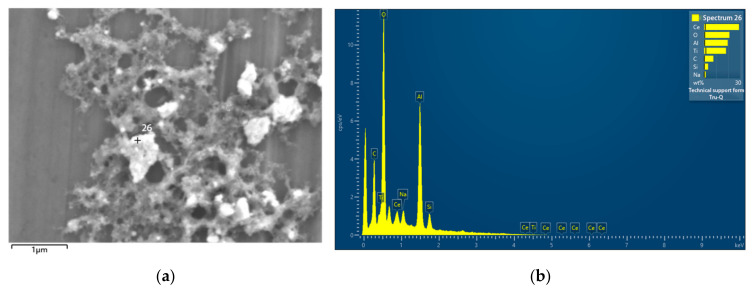
(**a**) SEM image of particles produced with 0.1 g ceria. (**b**) EDS spectrum of selected particle in (**a**). The crossing labeled with number 26 indicate the selected particle for EDS measurement.

Figure 13 presents EDS spectrum of a selected particle produced with 0.1 g ceria. It suggests that ceria became the majority and titania turned into the doping minority. It reveals that even in a very high diluted concentration 200 ppm in water, ceria was readily further broken down by the laser ablation, partially blocking the ablation of Ti target underneath.

## 4. Conclusions

In this study, we employed a laser marker for the fabrication of Ti-based nanoparticles (NPs) with and without the application of sonication. Notably, this is the first instance where sonication was utilized in laser nanoparticle fabrication. The introduction of sonication demonstrated several significant effects on the nanoparticle synthesis process. Firstly, sonication enhanced the production rate of NPs, as evidenced by ablated volumes extracted from confocal images. High frequency sonication may expedite the breakdown of the metastable cavitation bubble and plasma plume, accelerating the expulsion of NPs and increasing the production rate. Additionally, sonication resulted in a reduced particle size and a narrower size distribution, which was ascribed to be the stirring effect. The specific interaction mechanism between sonication and laser ablation is yet to be fully understood and requires further investigation. Moreover, particle size increased with laser fluence until a plateau fluence at 28.5 J/cm^2^. Furthermore, we found that a UV laser resulted in smaller particle size compared to the 1064 nm laser. The smallest nanoparticle size was estimated to be ~20 nm. It is worth noting that all particles exhibited a regular spherical shape and were predominantly oxidized, as confirmed by our analysis.

In addition, dispersing only 50 ppm ceria in water prior to laser ablation of Ti in water doped titania successfully. An amount of 200 ppm ceria in water led to titania doping of ceria. Further research is required to fully understand the underlying interaction mechanism between ceria, titania, and laser in order to optimize the fabrication process and tailor the properties of Ti-based nanoparticles for various applications.

## Data Availability

The data presented in this study are available on request from the corresponding author. The data are not publicly available due to privacy.
